# Challenges in the Prevention of Cervical Cancer in Romania

**DOI:** 10.3390/ijerph18041721

**Published:** 2021-02-10

**Authors:** Raluca Dania Todor, Gabriel Bratucu, Marius Alexandru Moga, Adina Nicoleta Candrea, Luigi Geo Marceanu, Costin Vlad Anastasiu

**Affiliations:** 1 Faculty of Economic Sciences and Business Administration, Department of Marketing, Tourism Services and International Affairs, Transilvania University of Braşov, Colina Universității Street, no. 1, Building A, Braşov 500068, Romania; raluca.todor@unitbv.ro (R.D.T.); gabriel.bratucu@unitbv.ro (G.B.); 2 Faculty of Medicine, Department of Medical and Surgical Specialties, Transilvania University of Braşov, B-dul Eroilor 29, 500036 Brașov, Romania; marceanu@gmail.com (L.G.M.); canastasiu@gmail.com (C.V.A.)

**Keywords:** cervical cancer, prevention, screening programs

## Abstract

Approximately every two hours, a Romanian woman is diagnosed with cervical cancer as the country ranks first in the EU in terms of its mortality rate. This paper aims to identify the main reasons that have led to this situation. First, a study based on secondary data was conducted in order to identify measures taken by the Romanian Ministry of Health for the prevention of this type of cancer. Second, a quantitative study was conducted to evaluate the impact that exposure to information and awareness campaigns has on women’s behavior regarding cervical cancer prevention through screening. The results of the research show an increased percentage of the women understanding the importance of screening and the benefits of early diagnosis, but also shows that a high percentage of women postpone the routine checks due to lack of time and financial resources. The research results also indicate that the only free screening program implemented in Romania during 2012–2017 was a failure due to poor procedures, low number of women tested, underfunding and the lack of promotion. Our conclusion is that the Romanian Ministry of Health has to take immediate action by conducting major awareness campaigns and also by implementing functional screening programs.

## 1. Introduction

In Europe, cervical cancer is the second leading cause of death in women, registering almost 24,000 deaths annually [1]. Worldwide, nearly 500,000 women develop this type of neoplasia each year, with most occurring in less developed countries where appropriate screening programs are not available [2]. The incidence of cervical neoplasia varies among countries. The highest incidences based on the age-standardized incidence rate (ASIR) per 100,000, calculated in 2018, are all found in countries from eastern, southern, or western Africa [3]: Swaziland (75.3), Malawi (72.9), Zambia (66.4), Zimbabwe (62.3) and Tanzania (59.1).

The World Health Organization (WHO) has stated that ‘regardless of the test used, the key to an effective program is to reach the largest proportion of women at risk with quality screening and treatment. The primary barrier to decrease the morbidity and mortality of this disease remains lack of awareness of the risk factors, causes, signs, and symptoms of cervical cancer’ [4].

### 1.1. Cervical Cancer. General Considerations

Human papillomavirus (HPV) infection is the leading cause of cervical cancer, even though the risk associated with different HPV types has not been adequately assessed [5]. Worldwide, the prevalence of HPV infection in cervical cancer is almost 99.7%. The presence of this virus in the majority of cervical neoplasias implies the highest fraction so far reported for a specific cause of human cervical cancer. Also, the rarity of HPV-negative cases reinforces the rationale for HPV testing in addition to cervical cytology examination [6].

Infection with high-risk HPV strains is a necessary, but not sufficient reason for cervical cancer [7]. Other identified risk factors involved in the development of cervical cancer are: smoking, early age at first intercourse, early age at first birth, low educational level and number of sexual partners. Meanwhile, a previous cesarean section is considered a protective factor [8].

Cervical cancer is preceded by precancerous lesions, which according to the current consensus can be divided into low grade squamous intraepithelial lesions (L-SILs) and high grade squamous intraepithelial lesions (H-SILs) [9]. The Pap smear test is a screening test for cancer and preinvasive lesions too, but abnormal results should be followed by colposcopy, biopsy and histopathological exam. Whereas low-grade squamous intraepithelial lesions usually heal spontaneously [10], and even high-grade lesions regress in up to 40% of cases [11], the 30-year progression risk of invasive cancer is 30% to 50% for untreated high-grade disease [12].

The progression from HPV infection to cancer occurs in four steps, as follows: HPV transmission, acute HPV infection, precancerous changes and finally, invasive cervical cancer. HPV type 16 and HPV type 18 are responsible for almost 70% of the cervical neoplasia cases [6]. Whereas HPV infection and abnormal findings of the cytology are normal among young women, invasive cervical cancer is rare in young females [13]. According to U.S. cancer statistics, only 0.2% of incident cervical cancer cases occur in women younger than 20 years, whereas 19.5% occur in women aged 65 years or older [6]. Vesco et al. [13] found that incident cases of cervical cancer in the United States seem to peak among women in their 40s, with approximately one half of incident cancer cases occurring in women 35 to 55 years of age.

The prognosis of cervical carcinoma depends on clinical (stage of the disease) and pathologic features (depth of invasion, lymph nodes invasion, lymphovascular space invasion, tumoral grading). In addition, some molecular markers such as the HPV strain may reflect the underlying biological bases for therapeutic management [14].

Indirect evidence indicates that cervical cancer screening should reduce the incidence and mortality of this neoplasia by about 90%. Annual screening is indicated, but the screening every 1 or 2 years compared with every three years improves the effectiveness by less than 5%. In the absence of screening, a woman has about 250:10,000 chances to develop invasive cervical cancer during her life, and about 118:10,000 chances of dying from this cause [15]. There are two types of screening for cervical cancer: Pap smear tests and HPV tests, which identify HPV strains. If high-risk HPV types are not detected, the risk to develop cervical cancer is low, and the woman can return for a new screening within 3 to 5 years [16]. The most powerful way to mitigate the damage inflicted by cervical cancer is through prevention, with increased efforts to educate communities on the risk factors and the need for screening and HPV vaccination [17]. Pap smear tests associated with medical treatment have resulted in substantial decreases in the burden of cervical cancer in Western and Northern Europe, but in Eastern Europe, the incidence and mortality of cervical cancer remain high.

Despite the fact that cervical cancer is a preventable type of cancer, Romania’s situation is extremely alarming. In Romania, cervical cancer is the third most common cancer in women, after breast and colorectal cancer [18], and the country occupies the first place in the EU regarding the mortality rates due to cervical cancer, according to the WHO [19]. With a mortality rate of 11.23/100,000, Romania’s situation is comparable to that of India (12.46/100,000) and several countries in South America such as Peru (11.61/100,000), Colombia (10.69/100,000) or Ecuador (12.97/100,000) [20]. Out of the total cases of cervical cancer diagnosed in the EU, 7.5% come from Romania, its rate being three times higher than the EU average [21].

The Romanian Ministry of Health implemented a free national screening program for five years in 2012. The results, however, were not in line with the expectations. A major barrier in the reduction of cervical cancer screening in Romania was the lack of a national cancer registry to monitor and record individual women’s screening history [22]. In Romania, laboratories and the doctors are not obliged to report the screening results to Romania’s regional cancer registries [23].

Considering the high mortality rates of cervical cancer in Romania, this study mainly aimed to identify the causes that have led to this situation. We also aimed to identify possible solutions to improve the situation of Romania from this point of view, while at the same timing reducing both financial and social costs.

### 1.2. Romania’s Situation among EU States

Romania is a developing country, located in the Southeast of Europe, which became a member of the EU in 2007. The population of Romania is currently estimated at 19.7 million inhabitants, with approximately 10.09 million women representing about 51.2% of the total population [24]. In terms of life expectancy, Romania ranks in 71st place in the world, with 71.4 years for men and 78.8 for women. Life expectancy in Romania is among the lowest in the EU and, although it has increased since 2000, it remains almost six years below the EU average [25]. The main causes of death among the population are heart diseases with a mortality rate of 212.34/100,000 (22nd place in the world), myocardial infarction 109.28/100,000 (58th place), lung cancer 30.56/100,000 (15th place), liver disease 26.75/100,000, breast cancer 19.5/100,000 (60th place), cervical cancer 11.23/100,000 (71st place). The mortality caused by all types of cancer represents the second cause of mortality in Romania, with 148.84 deaths per 100,000 people, placing our country in the 20th place in the world [26]. The five main types of cancer leading to death in Romania are lung, breast, colon, prostate and cervical cancer [26].

Romania, like other EU developing countries, has many areas concerning public health that still need to be developed. Remarkable contrasts were observed on the mortality rates of cervical cancer, particularly between the oldest and newest member states. These inequalities could be explained by the differences between the preventive strategies adopted during time [27]. Romania has, by far, the highest rate of mortality for cervical cancer in EU [28]. The EU average mortality rate due to this type of cancer is 3.4 per 100,000 inhabitants, while in Romania, approximately 11 women per 100.000 die every year of this disease [20].

Previous studies [29] have demonstrated the positive impact screening programs have had in various states of the EU. Those studies also showed that the situation of Romania is similar to that of other states in the 1960s and 1970s, before the existence of national screening programs. Those countries were facing high rates of mortality, but after the implementation of the cervical cancer screening programs, the mortality decreased significantly [30]. The importance of screening for the early diagnosis of cervical cancer and the fact that this increases the chances of curation [28], were highlighted in the Introduction section.

For a better understanding of the discrepancy between the average rate of mortality in the EU and Romania, it is necessary to analyze the situation of other member states of EU, regarding the measures taken to reduce the mortality rate caused by cervical cancer.

Austria introduced the first screening program in 1970 and during five decades the death rate for cancer reached 2.27/100,000. Approximately 1.5 million Pap smear tests are performed per year, exclusively by gynecologists. The cervical cancer mortality rates in this country decreased to one third during the past 40 years [31].

Belgium implemented the first program in the 1970s, and dramatically reduced the mortality rate from 15 to 5 deaths/100,000 women during this period. Due to the continuity of these programs, the current mortality rate has dropped nowadays to 2.10/100,000 women. The screening for cervical neoplasia between 1998 and 2000 included almost 59% of the Belgium female population, targeting the age group of 25–64 years old women. The number of performed tests was sufficient to cover more than 100% of the targeted population [32].

The Czech Republic reduced the mortality rate from 19.1 to 3.6/100,000. The first program was implemented in 1966, and it included one free gynecological examination and a Pap smear test annually. This right offered to the female population in this country is still maintained [33].

During the last 45 years, Germany decreased the rate of cervical cancer deaths by 75%. The first screening program for women was implemented in 1971 and the rate now is 2.06 deaths/100,000 women.

Although Italy implemented its first national screening program in 1996, rather late, compared to other countries, it has managed to reduce the mortality rate to only 1.56 deaths per 100,000 women.

Latvia is a very interesting case regarding the impact of these programs, as well as the importance of their continuation. In 1963, the mortality rate due to cervical cancer was alarming, 31.7/100,000. The screening programs implemented between the years 1970 and 1989 brought remarkable results, the rate falling to 8.9/100,000. In 1989, the program was discontinued and over the next 15 years the mortality rate increased to 19/100,000. In 2007, the screening program was re-launched, and all women over the age of 18 were screened. Today the mortality rate has dropped again to 6/100,000.

Like other developed countries in Western Europe, France started screening for cervical cancer in the 1970s, having today one of the lowest death rates in the EU, at 2.05/100,000. Currently, in France, screening for cervical cancer is free, and approximately 60% of the female population undergo a regular Pap smear test. Furthermore, this rate has increased to up to 80% in the younger population [34].

In Bulgaria, as in Romania, it is a sharp contrast in the cervical cancer mortality rates in comparison to Western countries [30]. The state had an initiative for such a program during the 2000–2006 period. The female population aged between 20 and 65 years old was to have been tested, but the program was never launched. Since then, there have been other initiatives, but these have also remained at the level of a plan. Thus, Bulgaria has the second, highest rate of deaths caused by this disease in the EU, with 7.04/100,000 [35].

On average, the proportion of women in EU countries aged 20–69 years who have been screened for cervical cancer within the past three years has increased from 56% to 61% over the past decade. However, the proportion has fallen in several countries. The proportion of screened women across EU countries still varies widely, from about 25% only in Latvia and Romania to over 80% in Austria and Sweden [36].

## 2. Materials and Methods

Starting from the main purpose of this paper, two types of study have been carried out. The first study was based on secondary data. These data were collected from freely accessible statistical sources provided by the WHO, the European Commission and the Ministry of Health in Romania.

The main objective was to understand the situation of Romania, by placing it in the EU context, and to identify the most important measures of prevention that the Ministry of Health has taken. Also, we aimed to evaluate the effectiveness of these measures.

In addtion we carried out a quantitative study on a group of 1945 Romanian women, aimed at identifying their attitudes and behaviors regarding screening for cervical cancer. The group consisted of females from urban areas, all of them belonging to the biggest community of mothers in Romania, known as “La Primul Bebe”. During the last 5 years, this community has carried out several campaigns regarding the importance of prevention through annual screening for cervical cancer.

This woman’s group was founded in 2012 and represents the largest community of educated women in Romania, with approximately 100,000 members. It is the only Romanian community awarded and supported by Facebook in 2018, together with other 99 communities from all over the world, who were considered to have a strong impact on society. In total, 6000 communities entered the competition and only 100 were selected to be part of the Facebook Community Leadership program [24]. One of the remarkable things this community has done is the ongoing campaigns to inform and empower members about the importance of preventing cervical cancer through screening.

A recent study that targeted the female population of Romania [37], showed that education plays a key role when it comes to women’s behavior towards screening investigations. This study demonstrates that the rate of women with higher education that undergo cervical cancer screening regularly is about four times higher than in the case of women with lower education levels. The study also highlights that overall, Romania’s female population has the lowest participation in screening investigations in the EU.

The main objectives of this part of the research were:

To measure the level of information of the subjects regarding the existence of the only screening program that was organized by the Ministry of Health in Romania from 2012 and 2017
To measure the level of awareness about the screening programs
To measure the degree of access to the screening programs
To identify the main reasons for which women decide to undergo a Pap smear test (regardless of the existence of a screening program)
To identify the main reasons for which women decide not to undergo periodical Pap smear tests
To measure the frequency of Pap smear tests among women.

The data from the respondents were collected through a survey that was distributed online through social media channels. Table 1 below presents the structure of the included sample by age, level of education and level of income.

The total number of participants was 1945. The sampling was achieved using a non-randomized conventional method. The participants are women from 14 large cities in Romania: Bucuresti, Ploiesti, Brasov, Cluj, Iasi, Constanta, Timisioara Galati, Braila, Craiova, Sibiu, Bacau, Pitesti and Suceava. Data were collected between October 2018-March 2019 using a Google Form questionnaire and the analysis was carried out using Microsoft Excel.

## 3. Results

**Figure 1:**
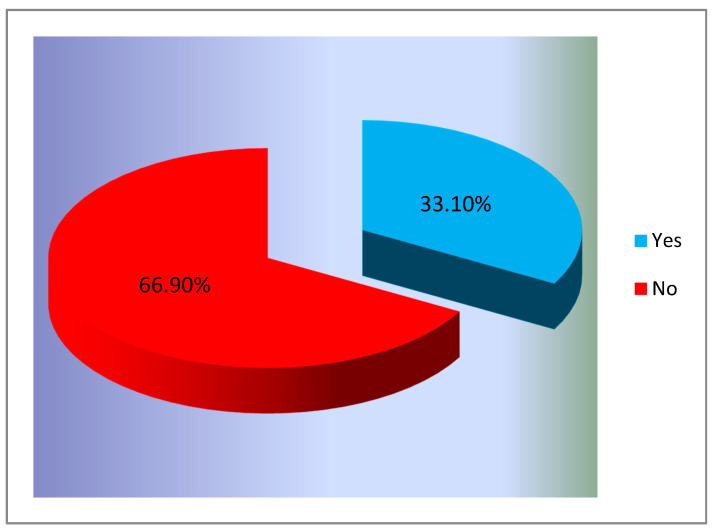
Respondents’ level of awareness about the existence of the Romanian cervical cancer screening program (% of total number of respondents, *n* = 1945).

The purpose of this study was to analyze the opinions and behaviors of educated Romanian females regarding the prevention of cervical cancer through regular screening. First, we measured the level of information the subjects had, regarding the existence of the only screening program that was organized by the Ministry of Health in Romania. Figure 1 presents the situation of the answers to the question “*Did you know about the existence of the screening program for cervical cancer, carried out during 2012–2017 in Romania*?”

The results showed that many participants did not know about the existence of this program. Only 644 women (33.1%) claimed to know about the existence of this program.

**Figure 2:**
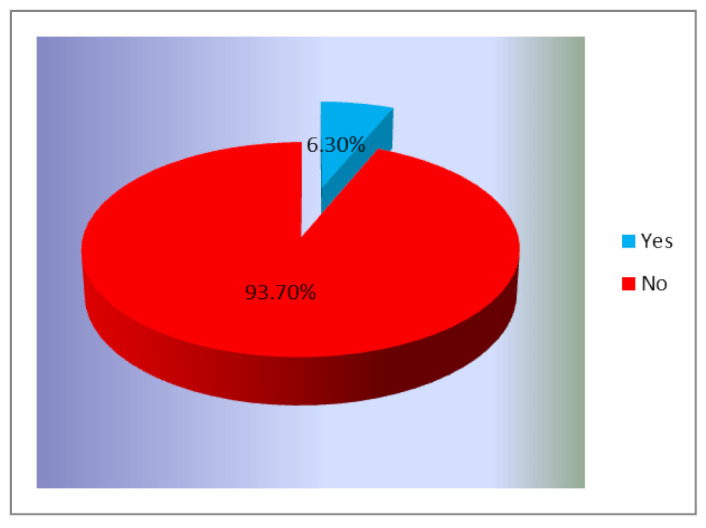
Respondents’ participation in the Romanian cervical cancer screening program (% of total number of respondents, *n* = 1945).

The results, presented in Figure 2, showed that an insignificant percentage of the investigated sample had benefited from free screening for cervical cancer during that period.

It can be seen that the participation rate was extremely low, as only 6.3% (122 women) benefited from free screening during this period. This is not entirely surprising, because if we correlate the low awareness of the existence of this program with the low rate of involvement from general practitioners in recommending the screening, this result was predictable.

**Figure 3:**
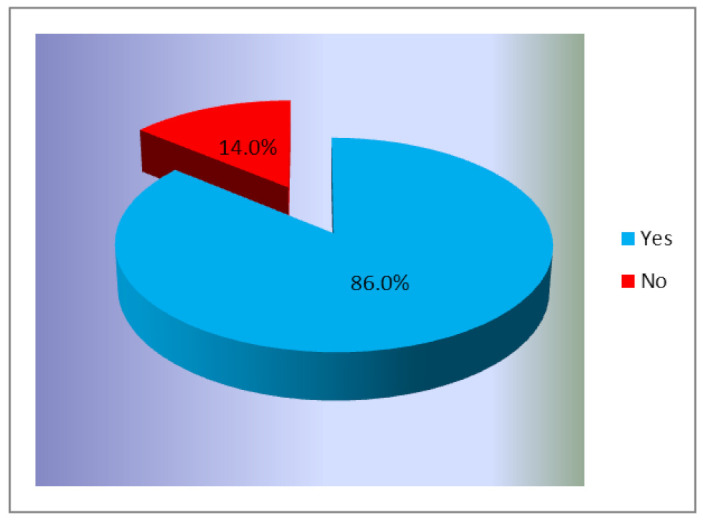
Respondents’ participation in a routine gynecological including a Pap smear test (% of total number of respondents, *n* = 1945).

Women were also asked if they “regularly see a doctor to perform a routine gynecological check that also includes a Pap smear test”. Figure 3 shows how women responded to this question.

The obtained results show that a large part of the participants, 1672 women (86%) are accustomed to regularly see a doctor for performing a routine check that also includes a Pap smear test. This fact reflects the impact that the information and awareness campaigns within the group had on the prevention behavior of these women. In addition, the fact that the researched group consisted of participants with higher education may explain this high number of women that understand the importance of the prevention.

**Figure 4:**
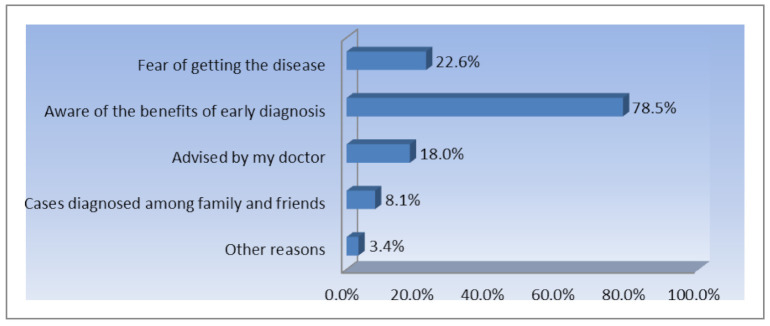
The main reasons motivating respondents’ cervical cancer screening (% of total number of respondents, *n* = 1672).

In order to better understand the behavior of the participants they were asked questions about the reasons why they choose to do/or not to do a routine Pap smear test. The main reasons for which women decide to make a screening check for cervical cancer through Pap smear test, are shown in Figure 4.

**Figure 5:**
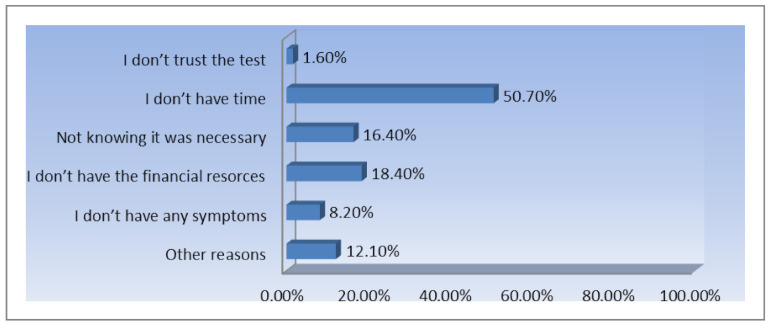
The main reasons causing respondents’ non-attendance to cervical cancer screening (% of total number of respondents, *n* = 273).

The results show that the most important reason is the awareness of the ”*benefits of early diagnosis*”—78.50% are motivated by this reason. This is another proof of the impact of the exposure to more information regarding the importance of prevention. Another explanation of this situation is that the participants are women living in urban areas, with high levels of education that improved their medical education as well. Afterwards, the “*fear of getting the disease*” seems to be important as well, 22.6% of the respondents admitting that this is the reason for which they performed routine tests. A small number of women responded that their doctor advised them to perform routine checks for this type of cancer just 18% were motivated by their doctors to make periodic Pap smear test. Among the most interesting answers provided by the subjects of the research were: “*I have had cervical cancer before*”, “*I am a gynecologist and I fully understand the need for annual testing*”, “*I have HPV infection, which developed after the pregnancy*”. Even more important was to find the motivation for which women choose not to present to screening Pap smear tests (Figure 5).

As Figure 5 illustrates, 50.7% of the women base their decision to not to attend periodic tests on a lack of time. This represents a problem because the impact of awareness raising campaigns is diminished in this case. The lack of financial resources is another important reason for which 18.4% of the participants choose not to undergo periodical Pap smear tests. This underlines the importance and need of implementing free national screening programs. Among the most interesting free answers received are the following: “*I am trying to prevent diseases through healthy eating*”, “*Out of convenience*”, “*I am afraid of the result*”, “*I simply forget*”, “*gynecological checks are very unpleasant*”, “*I am aware of the importance of the test but I always postpone doing it*”, “*Negligence, any other reason is just a pretext*”.

**Figure 6:**
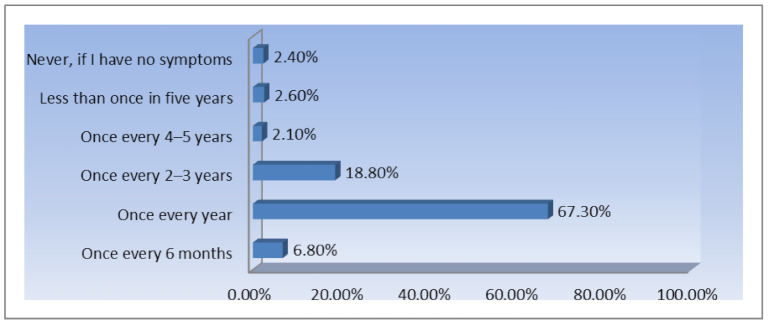
The frequency of routine Pap smear tests (*n* = 1945).

An important result of this study relates to Romanian females participation in cervical cancer screening. Figure 6 illustrates how often the respondents are taking routine Pap smear tests.

The research findings show that over 67% of the respondents (from a total of 1945 interviewed females) are taking a routine Pap smear test annually, while almost 19% are taking it every 2–3 years. However, these optimistic results need to be interpreted in the context of this study and considering the profile of the participants: educated females which have been exposed to awareness raising campaigns within the last 5 years, on the social media group “La Primul Bebe”.

## 4. Discussion

The Health System in Romania is based on the principle of contribution and does not offer differential medical services correlated with the amount of the contribution. The main objective of the Ministry of Health in Romania is “*a health system that supports and offers to the citizens the opportunity to achieve a better health, and that contributes to the increasing of the quality of their life*” [38]. Specific objectives include: “*improving population access to health care*”, “*increasing life expectancy*”, ”*the decrease of maternal and infant mortality*”, “*prevention of major infectious diseases* (TB, HIV) *as well as five chronic pathologies that can be early diagnosed and treated: cancer, cardiovascular diseases, diabetes, mental disorders, rare diseases*” [38]. However, many of these objectives remain at the declarative level and are not achieved in reality due to the lack of necessary measures.

Although the Romanian Ministry of Health understood the positive impact that a free national screening program could have in reducing the incidence and the mortality rates of cervical cancer, those screening programs are either not implemented yet, or they are, but the results have not been the expected ones.

Such an example was the first national cervical cancer screening program implemented by the Ministry of Health in 2012–2017 [39]. The specific objectives were: (1) the detection of cervical cancer in early stages, (2) guiding patients with precursor or incipient lesions to specialized medical services for diagnosis and treatment, (3) increasing the information level of the population, for the use of screening services as a method of early detection of cervical cancer in asymptomatic females. The research showed that the results of this five-year program were not the expected ones, and unfortunately, the mortality rates were not influenced. The initial goal of this program was to test 6 million women aged between 25–64 who did not have a confirmed diagnosis of cervical cancer [40]. By the end of the screening program, a little over 260,000 women were actually tested. An analysis showed that the main reasons for the lack of success of this program were [40]:

The program did not have national coverage and the penetration in the rural area was almost non-existent.

The lack of the promotion campaigns through mass media channels, in order to ensure the efficient transmission of the message about the existence of this program.

Only about 60% of the general practitioners participated in the program and 48% of them actively promoted this program and recruited patients.

The procedures were complicated and discouraged the women to enroll in this program. A general practitioner from Cluj reported: “*Although we recommended the patients to take this free routine Pap smear test, some of them refused because of the complicated procedures. Testing involved a difficult circuit of documents*”.

The program received little funding. The funding was completely stopped during certain periods. A general practitioner from Brasov reported: “*At one point I stopped recommending the patients to participate on the program, because there were no funds, it was a delicate situation and the credibility of the program has been affected*”.

Lack of program monitoring. There was no institution designated to monitor in real time the manner and quality of the program implementation, as well as the quantification of the number of tested women.

Considering this, WHO underlined the need of implementation of a new functional screening program in Romania that will be properly promoted and will provide easy access for a significant number of women to perform the Pap smear tests.

An optimistic finding of this study is that many subjects regularly see a doctor and perform a routine check, including Pap smear test. As few respondents mentioned, they were advised by their doctor to perform routine checks. An important measure which should be adopted in Romania concerns in stimulating gynecologists and general practitioners to inform and advise their patients regarding the necessity and benefits to periodically perform Pap smear tests.

The two main reasons invoked by those who do not make routine Pap smear tests were the lack of time and financial resources. Gizaw et al. [41] reported that the majority of women usually claim the lack of time as the most important issue used to justify their indifference. This personal motivation for not attending the screening for cervical cancer may be overcome through extensive educational programs. These initiatives may be started by various NGOs in partnership with the governmental medical authorities. Gynecologists and general practitioners also play an important role in the transmission of these messages regarding the importance of the screening. The financial motivation was also mentioned in the previous studies [42]. In our opinion, the reduction of the bureaucracy in all the processes needed for the implementation of such national screening programs is the key-point for a successful national screening program.

## 5. Conclusions

Considering the high rates of mortality for cervical cancer in Romania, this study investigated the potential causes that have led to this situation. The results of this study indicated that most of the investigated women did not know about the existence of the first national screening program for cervical cancer. A WHO report indicated the need to implement a new functional screening program in Romania for cervical cancer that should be properly promoted and should provide easy access for a wide range of females.

The first national screening program for cervical cancer implemented by the National Ministry of Health between 2012 and 2017 did not fulfil its initial purpose (testing 6 million females) and did not succeed in reducing mortality rates caused by cervical cancer in Romania. Moreover, the results of this study outline indicated that the interviewed Romanian women had little awareness about the existence of the Romanian cervical cancer screening program and therefore few had participated in this national screening initiative (122 respondents—6.3% of the total sample). Mainly motivated by the fear of getting the disease and by the awareness regarding the benefits of early diagnosis, most respondents acknowledged that they regularly see a doctor to perform a routine gynecological check that also includes a Pap smear test. However, this finding need to be interpreted within the context of the study’s sample, consisting of informed women from urban areas, who are members of the largest community of educated mothers in Romania, and as the results show, have above average incomes, which allows them to pay the cost of the Pap smear tests. The main reasons causing respondents’ non-attendance to cervical cancer screening (lack of time and of financial resources) point out once again the importance and need of implementing free national screening programs, which need to be intensively promoted among Romanian females from both urban and rural areas, accompanied by awareness raising campaigns regarding the incidence and mortality of cervical cancer in the country.

Furthermore, the results of this study need to be contextualized in the current Covid-19 pandemic that has provided the opportunity to find solutions to overcome some barriers related to HPV vaccination, education and counseling and the use of primary HPV screening [43] The huge financial losses suffered by all medical systems around the world in the context of the current pandemic should not be an obstacle to the further development of effective screening programs such as cervical cancer one. Despite the obstacle of patients’ reluctance to visit clinics and the postponement of health issues that do not seem urgent, the positive aspect is a clearer awareness of the benefits of telemedicine, the possible orientation of patients towards self-sampling for HPV and increasing adherence to vaccination in general [43].

Although this study provides an overview on the situation of Romania regarding cervical cancer, it has some limitations, which pave the way for further studies. The main limit is represented by the sample selection method, which was non-randomized. However, the large number of participants from 14 major cities of Romania, has diminished the disadvantage regarding the sampling method. Another limitation is represented by the exclusion of the subjects from rural areas, because the screening program developed between 2012–2017 did not target the rural area.

Future research directions should refer at conducting a more extensive research on the female population of Romania, which will also include subjects from the rural areas and women with low to medium educational level.

## Figures and Tables

**Table 1:** Sample structure by age, educational and income level.

Characteristic		Number of Women Percent
Age	20–30	574 29.5%
	30–40	1310 67.3%
	40–50	55 2.8%
	50–60	6 0.4%
Total		1945 100%
Level of education	Gymnasium	- 0%
	High School	93 4.8%
	Bachelor’s Degree	991 50.9%
	Master Degree	861 44.3%
Total		1945 100%
Level of income	1000–1500	191 19.4%
	1500–2000	325 27%
	2000–3000	533 23.4%
	3000–4000	263 10.1%
	4000–5000	232 9%
	Over 5000	401 11.1%
Total		1945 100%

## Data Availability

The data presented in this study are available on request from the corresponding author. The data are not publicly available due to privacy reasons.
